# Kynurenines with Neuroactive and Redox Properties: Relevance to Aging and Brain Diseases

**DOI:** 10.1155/2014/646909

**Published:** 2014-02-17

**Authors:** Jazmin Reyes Ocampo, Rafael Lugo Huitrón, Dinora González-Esquivel, Perla Ugalde-Muñiz, Anabel Jiménez-Anguiano, Benjamín Pineda, José Pedraza-Chaverri, Camilo Ríos, Verónica Pérez de la Cruz

**Affiliations:** ^1^Departamento de Neuroquímica, Instituto Nacional de Neurología y Neurocirugía Manuel Velasco Suárez, S.S.A., Insurgentes Sur 3877, 14269 México, DF, Mexico; ^2^Área de Neurociencias, Departamento de Biología de la Reproducción, Universidad Autónoma Metropolitana-Iztapalapa, 09340 México, DF, Mexico; ^3^Laboratorio de Neuroinmunología, Instituto Nacional de Neurología y Neurocirugía Manuel Velasco Suárez, S.S.A., 14269 México, DF, Mexico; ^4^Departamento de Biología, Facultad de Química, Universidad Nacional Autónoma de México, 04510 México, DF, Mexico

## Abstract

The kynurenine pathway (KP) is the main route of tryptophan degradation whose final product is NAD^+^. The metabolism of tryptophan can be altered in ageing and with neurodegenerative process, leading to decreased biosynthesis of nicotinamide. This fact is very relevant considering that tryptophan is the major source of body stores of the nicotinamide-containing NAD^+^ coenzymes, which is involved in almost all the bioenergetic and biosynthetic metabolism. Recently, it has been proposed that endogenous tryptophan and its metabolites can interact and/or produce reactive oxygen species in tissues and cells. This subject is of great importance due to the fact that oxidative stress, alterations in KP metabolites, energetic deficit, cell death, and inflammatory events may converge each other to enter into a feedback cycle where each one depends on the other to exert synergistic actions among them. It is worth mentioning that all these factors have been described in aging and in neurodegenerative processes; however, has so far no one established any direct link between alterations in KP and these factors. In this review, we describe each kynurenine remarking their redox properties, their effects in experimental models, their alterations in the aging process.

## 1. Kynurenine Pathway

**Figure 1:**
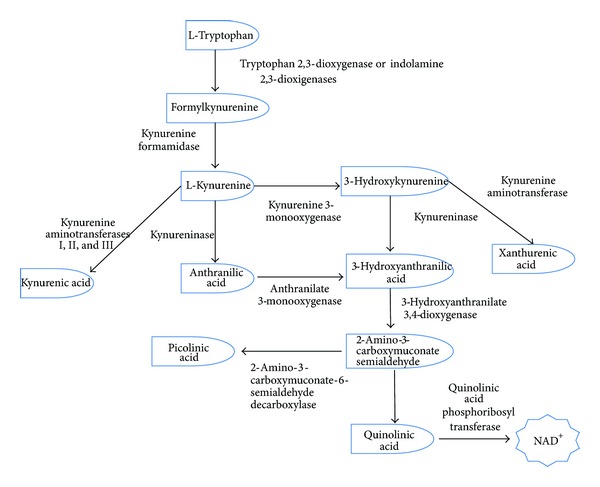
Kynurenine pathway.

The main route of catabolic tryptophan degradation is through kynurenine pathway (KP) which leads to production of nicotinamide adenine dinucleotide (NAD^+^; Figure 1) [1]. This pathway takes place mainly in the liver, kidney, and brain of humans, primates, rodents, and other small mammals [2]. It is noteworthy that humans and animals do not possess the enzymatic machinery to synthesize tryptophan by themselves, the reason why they get tryptophan from the diet. The KP is particularly modulated by the regulatory mechanisms of the immune response and by the redox status. The metabolites most widely studied are kynurenic acid (KYNA) and quinolinic acid (QUIN) due to their neuroactive capacities, while indoleamine dioxygenase-1 (IDO-1), 3-hydroxykynurenine (3-HK), and 3-hydroxyanthranilic acid (3-HA) are studied mostly due to their redox properties and modulation.

The first step of the KP involves the oxidative opening of the tryptophan indole ring by tryptophan 2,3-dioxygenase (TDO; in the liver) or by indoleamine 2,3-dioxygenase-I and -II (IDO-1 and IDO-2, resp., in the brain) to produce the instable metabolite, N-formylkynurenine [3–5]. The next step is the conversion of N-formylkynurenine to L-kynurenine (L-KYN), a metabolite that will serve as substrate for various enzymes: kynureninase which produces anthranilic acid (ANA), kynurenine aminotransferases (KAT I, II, and III), that catalyze the irreversible transamination from L-KYN to kynurenic acid (KYNA), and kynurenine 3-monooxygenase (KMO) that catalyzes the synthesis of 3-hydroxykynurenine (3-HK). Then 3-HK can be taken by kynurenine aminotransferase (KAT) to produce xanthurenic acid (XA) or by the kynureninase to form 3-hydroxyanthranilic acid (3-HA), which can also be produced by ANA through anthranilate 3-monooxygenase.

3-Hydroxyanthranilate dioxygenase (3-HAO) opens the ring of 3-HA to produce 2-amino-3-carboxymuconate semialdehyde, an unstable intermediate which is immediately transformed into QUIN. Finally, quinolinate phosphoribosyltransferase (QPRT) produces NAD^+^ from QUIN [6].

## 2. Enzymes Modulated by Redox Status

The flux through the KP in brain is rate limited by IDO, a cytoplasmic enzyme that converts tryptophan to the catabolism products collectively known as kynurenines [7]. IDO is a heme enzyme found in the central nervous system (CNS) which has high affinity for L-tryptophan (Km ~ 0.02 mM) and requires oxygen [8, 9] for its activity. However, IDO-1 kinetically prefers superoxides instead of oxygen [10] and can use them both as substrate and as cofactor. In fact, one of the suggested roles for IDO-1 is that it can act as scavenger of superoxide (Table 1) [11]. This function is due to the ability of superoxide to reduce inactive ferric IDO-1 to the active ferrous form [12]; then takes place the oxidation of the pyrrole ring of tryptophan to form N-formylkynurenine. IDO-1 becomes more active with increasing oxygen concentrations and, *in vivo*, KYN is 60% higher in brains of HBO-convulsed rats compared with rats breathing air. The intracellular reducing co-factor(s) of IDO-1 include(s) superoxide anion, dihydroflavin mononucleotide, tetrahydrobiopterin, and cytochrome reductases [12, 13]. IDO-1 can be directly activated by a number of cytokines, including IFN-*γ* and TNF-*α*. This dioxygenase is present in accessory immune cells, including macrophages and dendritic cells, and it is expressed in all organs including brain [14, 15]. Hydrogen peroxide and oxide nitric are inhibitors of IDO-1 [12, 16]. Inhibition of IDO-1 by a competitive or a noncompetitive inhibitor resulted in a dose-dependent decrease in its activity which correlated directly with the decreasing intracellular NAD^+^, which causes decreased cell viability and CNS functions [17].

Another enzyme that participates in tryptophan (Trp) degradation through the kynurenine pathway is IDO-2 enzyme that is encoded by a homologous gene of IDO-1 [18, 19]. In humans, IDO-2 is expressed in placenta, brain, liver, small intestine, spleen, thymus, lung, kidney, and colon [19]. It seems that IDO-2 has lower activity than IDO-1 [18, 19] and its participation in L- Trp oxidation remains unclear since it has been shown, in some studies, that there is no detectable kynurenine formation *in vivo* associated with IDO-2. However, it has been related to an increase in KYN levels and IDO-2 expression, but not with IDO-1, in human carcinoma cells treated with the chemokine CXCL11 [20]. Additionally, it was described that IDO-2 showed lower Km than IDO-1 in different species (mouse: Km ≈ 29 *μ*M and 12 mM for IDO-1 and IDO-2, resp.) and both enzymes also differ in other biochemical properties such as pH and thermal stability [21]. Thus, although that has not been found a specific physiological role for this enzyme, it is apparently quite different to IDO-1. This evidence suggests that IDO-2 is active under specific conditions; therefore it depends on the presence of specific factors and the cell type [22].

KMO is another important enzyme; it is a NADPH-dependent flavin monooxygenase. This monooxygenase is localized in the outer mitochondrial membrane in the CNS and is predominantly expressed in microglia [23–26]. KMO exists as an apoenzyme and interacting with flavin-adenine dinucleotide (FAD) forms a holoenzyme; the flavin moiety of the protein acts as an electron donor [27]. The specific function of KMO is catalyzing the incorporation of one atom of oxygen into kynurenine, in the presence of NADPH as electron donor. During the reaction, the prosthetic group FAD is reduced to FADH_2_ by NADPH and subsequently oxidized by oxygen to FAD. Further kinetic studies have demonstrated that the enzyme activity could be inhibited by pyridoxal phosphate and Cl^−^ (Table 1) [28]. The relevance of KMO activity, in both physiological and pathological conditions, is that this enzyme possesses a high affinity for the substrate (Km is in the low micromolar range), thus suggesting that it metabolizes most of the available kynurenine to produce 3-HK [29]. Notably, it has been reported that KMO expression increases in inflammatory conditions or after immune stimulation [30]. Due to the alterations in the KP metabolites in various pathologies, the enzymes of this pathway represent significant targets for therapeutic intervention and KMO is one of the main enzymes studied.

Kynureninase is a pyridoxal phosphate-dependent enzyme, which is mainly located in the cytosol and catalyses the transformation of KYN into ANA as well as of 3-HK to 3-HA. It exhibits a 10-fold higher affinity for 3-HK than for KYN. The optimum pH of the enzyme is 8.25 and it displays a strong dependence on the buffer ionic strength for optimum activity [31]. Mn^2+^ ions activate kynureninase only in the presence of added pyridoxal phosphate, whereas Ca^2+^ ions activate it in presence and absence of added pyridoxal phosphate (Table 1) [32].

The enzyme that catalyzes the final aromatic ring opening reaction in the KP is the 3-HAO. In this enzymatic reaction 3-HA produces an unstable compound, *α*-amino-*β*-carboxymuconic *ε*-semialdehyde, which is then nonenzymatically transformed to QUIN. 3-HAO is present in small amounts, in mammalian brains [33], mainly in astrocytes surrounding glutamatergic synapses in the CNS [34]. For its activity, 3-HAO requires both nonheme Fe^2+^ to incorporate atoms of molecular oxygen into 3-HA and sulfhydryl groups [35, 36]. Recently, it was demonstrated that Fe^2+^ stimulates 4- to 6-fold 3-HAO activity, in striatal homogenates of mouse, rat, and human; this effect is prevented by ferritin [37].

On the other hand, QPRT has been identified in rat and human CNS [38]. Magnesium ions are required for QPRT activity and there is evidence that a cysteine residue at the active site is required for catalysis [39]. Interestingly, QPRT is in a P2 synaptosomal fraction particulate component [40]. This enzyme is particularly important since it catalyzes the conversion of QUIN to NAD^+^; changes in the amount of QPRT protein alter the intracellular ratio between NADH/NAD^+^ and ATP; in consequence, QUIN is accumulated, promoting the excitotoxic damage.

The kynurenines aminotransferases (KATs) are key in the KP since they produce the only endogenous antagonist of NMDA receptor, KYNA. In mammalian peripheral organs, several rather unspecific pyridoxal-5′phosphate-dependent aminotransferases are able to catalyze the conversion of KYN to KYNA [41–44]. However, in the brain of humans, rats, and mice, four proteins (KAT I, II, III, and IV) seem to be responsible for KYNA production [35, 44–49], of which KAT I and KAT II are the most studied. KAT I prefers pyruvate as co-substrate [50] and it is strongly inhibited by the competing substrates such as tryptophan, phenylalanine and glutamine. Immunohistochemical studies in rat brain have demonstrated that this enzyme is located preferentially in astrocytes. KAT II has a slight preference for oxoglutarate as a cosubstrate and also displays L-aminoadipate aminotransferase activity. This enzyme is inhibited by *α*-aminoadipate and quisqualate. 3-HK inhibits both KAT I and KAT III activity but is more active against KAT II [44]. Currently, there are different crystallographic structures of KATs deposited in the Protein Data Bank (PDB), which allows us to give a structural interpretation into catalysis and inhibition mechanism of these enzymes.

## 3. Metabolites with Redox and Neuroactive Properties

### 3.1. Tryptophan

Trp is an essential amino acid, and its structure contains a ring that can stabilize radicals through resonance or delocalization, thus enabling it to break radical chain reactions [51]. Trp is able to react with hydroxyl radicals and to trap tert-butoxyl radical (CH_3_)_3_CO^•^, with rate constant values of *κ* = 10^10^ M/s and 2.8 × 10^9^ M/s, respectively [52]. Analyses performed with other indolic structures have shown that ONOO^−^ reacts preferentially with 3 substituted indoles such as Trp derivatives rather than with unsubstituted indoles; and the most important products observed at physiological pH are 1-nitrosotryptophan derivatives kynurenines/kynuramines obtained by opening of the pyrrole ring [53]. Moreover, the administration of Trp decreased the lipid peroxidation induced in rats under experimental endotoxic shock, suggesting antioxidant properties of this amino acid [54]. This finding is consistent with the report of Pazos and coworkers [55], who showed that Trp is the amino acid with the highest antiradical activity. In addition, tryptophan turned out to be a potent scavenger of radicals induced by chloramine T or hydrogen peroxide, which was detected by a chemiluminescence assay [56].

### 3.2. Kynurenine

A central compound of the KP is KYN, given that it is a substrate for different enzymes to produce KYNA, 3-HK, or ANA. Some reports have shown a protective effect of KYN in toxic experimental models. However, this effect has been attributed mainly to the production of KYNA, which has an antagonist effect on both NMDA and *α*7-nicotinic receptors. Nevertheless, KYN *per se* has scavenging properties that should be considered to explain the effects of this metabolite in the toxic models in which has been tested.

Zsizsik and Hardeland observed KYNA formation from KYN in light-exposed homogenates of the dinoflagellate *Lingulodinium polyedrum, *which was under a prooxidant environment induced by paraquat and CCCP, suggesting that oxidative kynurenine deamination leads to KYNA production; furthermore, in this process KYN could be acting as an antioxidant [57]. This finding correlates with the fact that L-KYN reduces the chemiluminescence induced by hydrogen peroxide or chloramine T [56] and also with its ability to trap hydroxyl radical (*K*_*r*_  1.4 × 10^10^ M^−1^s^−1^; determined by EPR-spin trapping and pulse radiolysis method) Table 2 [58, 59]. Recently, it has been showed that L-KYN was able to abolish ROS production induced by 3-nitropropionic acid and ONOO^−^; this effect was independent of KYNA formation since the samples were obtained from brain homogenates of KAT II knockout mice (which lack the major enzyme for the biosynthesis of KYNA) [60]. Altogether, this evidence strongly suggests that KYN can be considered as a potential endogenous antioxidant, which can donate an electron and protect macromolecules *in vivo *and *in vitro* against oxidative modifications [53, 61]. These properties can be independent of the KYNA formation.

However, KYN has also shown prooxidant effects. It has been described that aerobic irradiation of KYN produces superoxide radicals and leads to reduction of cytochrome c [62, 63]. Additionally, *in vitro *studies show that KYN is able to photooxidize cysteine, NADH, and ascorbic acid and this capacity may be directly relevant to photobiological processes occurring in the lens *in vivo*. In particular, these photooxidation processes can be responsible for the age-related depletion of reduced glutathione and/or formation of hydrogen peroxide in lens [64]. On the other hand, KYN can also cause cell death through ROS pathway in NK cells [65].

### 3.3. Kynurenic Acid

The major KP metabolite considered as neuroinhibitor is KYNA, which is synthesized and released by astrocytes and antagonizes NMDAr [66] and *α*7-nicotinic acetylcholine receptor (*α*7nAChR) [67]. KYNA synthesis is mediated by KATs. Studies in rodents have shown that modest increases or reductions in KYNA levels decrease or facilitate extracellular dopamine and glutamate release, respectively [68–72]. Accordingly, dysregulation of endogenous KYNA may contribute to the physiopathology of several disorders [73–75].

Recently, KYNA was identified as an endogenous ligand of GPR35 [76]. This fact highlighted the importance of KP in regulating immune functions because the activation of GPR35 inhibits TNF-*α* release by macrophages under inflammatory conditions induced by LPS. Upon this context, KYNA might exert an anti-inflammatory effect [76]. Additionally, GPR35 decreases intracellular Ca^2+^ probably by inhibiting its entrance [77]; thus, KYNA most likely exerts an effect on the release of inflammatory mediators and excitatory amino acids from glial cells. The ligand-activated transcription factor aryl hydrocarbon (AHR), a nuclear protein involved in the regulation of gene transcription, is also activated by KYNA and is able to cause immunosuppression [78].

On the other hand, various groups have studied the redox properties of KYNA. This kynurenine is a reducing agent that might even be able to act as (electron transfer) redox catalyst *in vivo*. KYNA has been shown to scavenge hydroxyl radicals; it is able to prevent radicals-induced malondialdehyde formation from 2-deoxyribose [79–82]. However, KYNA can behave, under certain circumstances, as a prooxidant since it has been shown to have a strong potentiation of the prooxidants properties of *δ*-aminolevulinic acid [83]. The putative mechanism by which KYNA scavenges free radicals was proposed by Zsizsik and Hardeland [82]; the reaction is initiated by the hydroxyl radical, and then a decarboxylation should be the favored process. The resulting decarboxylated cation radical interacts with another hydroxyl radical, and the next intermediate interacts with superoxide, leading to the nitric oxide release. The resulting 2-hydroxychromanone then may be in equilibrium with its tautomer, 2,4-dihydrochromene. The balance of the radical scavenging is that three radicals would be scavenged (two of OH^•^ and one of superoxide) and one would be formed (^•^NO) [82]. Additionally, another study showed that KYNA can also scavenge peroxynitrite. It also can prevent the lipid peroxidation and ROS production in rat forebrain homogenates and in *Xenopus laevis* oocytes (preparation free of NMDA receptors) induced by FeSO_4_, suggesting that the protective effect of KYNA is independent of its activity over receptors. An *in vivo* study also showed that KYNA decreases the hydroxyl radical formation induced by the acute infusion of FeSO_4_ in the rat striatum [84]. Furthermore, it has been shown that KYNA significantly increased oxygen consumption during state IV respiration leading to an impaired respiratory control index and ADP/oxygen ratio [85, 86].

All these evidences show that KYNA is an important neuromodulator but also is an endogenous antioxidant and its protective effect showed in divers toxic models may be due to its redox character in addition to its activity on receptors.

### 3.4. 3-Hydroxykynurenine (3-HK)

3-HK is a controversial kynurenine since it has shown prooxidants and antioxidants activities. The structure-toxicity relationship shows that the *o*-aminophenol structure common to 3-HK is required to exert its toxicity. *o*-Aminophenol compounds are considered to be subject to several steps of oxidation reactions initiated by their oxidative conversion to quinoneimines, which is accompanied by concomitant production of ROS, generating mostly superoxide anion and H_2_O_2_ (Table 2) [87].

The neurotoxicity of 3-HK in primary neuronal cultures prepared from rat striatum is blocked by catalase and desferrioxamine but not by superoxide dismutase, indicating that the generation of H_2_O_2_ is involved in the toxicity. The protective effect of desferrioxamine suggests a role for iron in 3-HK toxicity, either in catalyzing the oxidation of 3-HK or in promoting the reduction of H_2_O_2_ to the highly reactive hydroxyl radical. Additionally, it has been proposed that the ROS generation by low concentration of 3-HK (1–10 *μ*M) occurs intracellularly and depends on the 3-HK uptake activity which is variable in the different brain regions [88]. This is one of the possible mechanisms by which 3-HK induces cell death [89]. It has been showed that the endogenous xanthine oxidase activity is involved in the H_2_O_2_ generation produced by 3-HK and also exacerbates cell damage generated by this kynurenine. However, the precise mechanism by which this enzyme is acting in this process is not clear [89]. 3-HK, besides being considered as cytotoxic for neuronal cells [90], has also been shown to cause bladder cancer [91]. Moreover, 3-HK modifies the respiratory parameters, decreasing respiratory control index as well as ADP/oxygen ratio of glutamate/malate-respiring heart mitochondria [87].

On the other hand, it has been demonstrated that 3-HK and 3-HA reduce Cu(II) and both generate superoxide and H_2_O_2_ in a Cu-dependent manner [92]. The incubation of bovine *α*-crystallins with low concentrations of 3-HK causes protein cross-linking and oxidation of methionine and tryptophan residues [93], indicating that the protein damage likely results from generation of reactive oxygen species. In the human lens, these reactions have been associated with both aging [94] and cataractous processes [95]. Also, it was shown that 3-HK and 3-HA provoke protein oxidative damage and induce apoptosis characterized by chromatin condensation and internucleosomal DNA cleavage in PC12, GT1-7, and SK-N-SH cells [92, 96–98]. *In vivo *experiments have demonstrated that injection of 3-HK into the striatum causes tissue damage that is prevented by N-acetyl-L-cysteine coapplication [99].

Conversely, 3-HK has also been proposed to be an antioxidant, peroxy radicals scavenger in inflammatory diseases [100], and superoxide scavenger in the Malpighian tubes of insects [101]. Since 3-HK is an *o*-aminophenol, it might be expected to undergo complex oxidative processes. In fact, under severe oxidative stress induced via the hydrogen peroxide-horseradish peroxidase system, 3-HK forms hydroxanthommatin and xanthommatin, products of six- and eight-electron oxidations, respectively [87]. The initial stable product of autooxidation of 3-HK does react with O_2_^•−^ (lower limit for *k* is 5.6 × 10^6^ M^−1^ s^−1^), and it is possible that this autooxidation product could be responsible for protection from the deleterious effects of O_2_^•−^ [59]. The amount of 3-HK is abundant in Malpighian tubes of insects and was reported to work as a major antioxidant in the tubes [101, 102].

Besides, 3-HK and 3-HA, like vitamin C and Trolox, belong to the class of small molecules that react very rapidly with peroxyl radicals and hence are potentially important biological antioxidants. In particular, 3-HK and 3-HA protected B-phycoerythrin from peroxyl radical-mediated oxidative damage more effectively than equimolar amounts of either ascorbate or Trolox [100]. 3-HK was more reactive with the ferryl complex than glutathione, suggesting that the antioxidative efficiency is better than glutathione. Additionally, the C6 glioma cells exposed to 3-HK increased its total antioxidant reactivity values and the TBA-RS levels were decreased without changing the morphology of the cells [103].

This redox behavior of 3-HK can be explained by Giles and coworkers, who propose that 3-HK can initially act as two-electron donors (antioxidant) but the *ortho*-quinone-imine formed oxidatively and the ROS produced in this process are responsible for its prooxidant effects [104]. Therefore the behavior of 3-HK depends on the redox status of the cell.

### 3.5. Xanthurenic Acid

Xanthurenic acid (XA), a metabolite of the KP is synthesized through 3-HK transamination, and it is closely related structurally to KYNA but possesses different biological roles; actually the biological function of XA remains obscure. Gobaille and coworkers proposed that XA can have a role in the neurotransmission/neuromodulation since it is actively taken up by synaptic vesicles from rat brain, effect that is inhibited in absence of ATP [105].

Some groups have focused on the study of the redox properties of this metabolite, which have showed metal-chelating activities and antioxidant properties [106, 107]. Zsizsik and Hardeland showed that XA turned out to be an efficient scavenger of hydroxyl radicals and ABTS^•+^ produced in the ABTS system. XA was able to inhibit the lipid peroxidation induced by iron and copper oxidation in low density lipoprotein, and this metabolite also prevents the inactivation of NADP-isocitrate dehydrogenase produced by the oxidation of these metals [106]. XA scavenges superoxide in a hematoxylin autooxidation system [108]. XA has also been shown to act as a peroxyl radical scavenger *in vitro* [100]. A recent study evaluated the antioxidant action of XA using heme and iron as promoters of radical formation. In this model, XA proved to be a powerful antioxidant, inhibiting lipid peroxidation in a pH-dependent manner [109]. The antioxidant properties of XA could be related to the fact that all phenolic metabolites show antioxidant activities points toward the importance of the phenolic moiety as the active entity [100].

On the contrary, XA sometimes acts as a prooxidant due to its chelating effect. Recent studies revealed that XA binds ferric ion at the 8-hydroxyl group and the nitrogen atom of the quinoline moiety, resulting in the enhancement of the autooxidation of ferrous ion to ferric ion [110]. The formation of metal-chelate complex modifying the oxidation-reduction potential of metal ion is responsible for the generation of reactive oxygen species (ROS) [111]. Oxygen molecules accept one electron from ferrous ion to form superoxide radical, which can also produce another ROS. Once that XA forms the metal complex, inactivates aconitase through ROS generation mainly hydroxyl radical [112]. Furthermore, XA was demonstrated to act as an apoptosis-inducing metabolite in vascular smooth muscle and lens epithelial cells [113, 114]. Additionally, XA acts as a photosensitizer and generates superoxide and singlet oxygen upon irradiation [115]. The photooxidation and polymerization by XA of lens proteins are related to the age-dependent cataractogenesis [116, 117]. All these studies suggest that the cytotoxic action of XA may be explained by the prooxidant properties of chelate complexes with metals.

### 3.6. 3-Hydroxyanthranilic Acid

Many studies considered 3-HA as free radicals generator [28, 29] because in its autooxidation it is able to generate free radicals. This autooxidation of 3-HA involves first, the generation of semiquinoneimine (anthraniloyl radical) which oxidizes to the quinoneimine, followed by condensation and oxidation reactions to yield a cinnabarinic acid. 3-HA auto-oxidation to cinnabarinate requires molecular oxygen and generates superoxide radicals and H_2_O_2_. Superoxide dismutase (SOD) accelerates 3-HA auto-oxidation, probably by preventing back reactions between superoxide and either the anthraniloyl radical or the quinoneimine formed during the initial step of auto-oxidation. Mn^2+^, Mn^3+^, and Fe^3+^-EDTA catalyze cinnabarinate formation under aerobic conditions [118].

In experimental models, the pattern of 3-HA in mitochondrial processes involves the inhibition of oxygen uptake by mitochondrial respiring with NAD-dependent substrates, uncoupling the respiratory chain and the oxidative phosphorylation [87, 119]. A marked inhibition (79%) of oxygen uptake by 1 mM 3-HA was observed in an oxoglutarate-respiring rat liver or rat heart mitochondria [119]. Furthermore, it has been shown that 3-HA induces apoptosis in monocyte/macrophage cell lines, and the apoptosis response was enhanced by ferrous or manganese ions, according to a mechanism that presumably involves production of hydrogen peroxide, since the effect was attenuated by catalase [120]. Fallarino and coworkers [121] showed that both 3-HA and QUIN can induce apoptosis of thymocytes of terminally differentiated T helper cells, in particular, Th1 but not Th2 clones, through Fas-independent mechanisms involving the activation of caspase-8 and the release of cytochrome c from mitochondria. It has also been suggested that 3-HA inhibits NF-*κ*B activation upon T cell antigen receptor engagement by specifically targeting PDK1 [122]. Additionally, it was demonstrated that 3-HA induced depletion of intracellular glutathione in activated T cells without increased ROS formation [123].

On the contrary, there are also reports that show that 3-HA is a potent antioxidant [124] and downregulates the inducible nitric oxide synthase expression [125, 126] by enhancing OH-1 expression in macrophages stimulated with IFN-*γ* and lipopolysaccharide, thereby resulting in a further increase in IDO expression and activity [127]. Additionally, 3-HA reduces *α*-tocopheroxyl radical restoring the levels of *α*-tocopherol and preventing LDL lipid peroxidation [124, 128].

Furthermore, 3-HA and 3-HK inhibited the spontaneous lipid peroxidation in the brain and this inhibitory property remained even in the presence of Fe^3+^, protecting cerebral cortex against oxidative stress [129]. The GSH spontaneous oxidation and the peroxyl radicals were significantly prevented by 3-HA [103].

Electrochemical studies suggest that 3-HA can initially act as antioxidant and next as a prooxidant [104] since the *ortho*-quinone-imine formed possesses oxidant properties. The most likely explanation for the dual effect *in vitro* of 3-HA is a concentration-dependent action.

### 3.7. Anthranilic Acid

Although ANA is generally accepted to be biologically inactive, it can interact with copper to form an anti-inflammatory complex. This complex acts as a hydroxyl radical-inactivating ligand able to remove the highly injurious hydroxyl radicals at inflammatory sites. However, the ANA-Cu^2+^ complex increases the Fenton reactivity of copper, producing more hydroxyl radicals, which are quickly removed by the same complex [130, 131]. Due to this property, the synthetic derivative of ANA, 3-methoxyanthranilate, has been proposed as a potential anti-inflammatory drug [132].

Nevertheless, in a study *in vitro* using organotypic cultures of rat hippocampus it was demonstrated that ANA (at high mM concentration) may cause neurodegeneration [133]. However, the mechanism of this finding has not been elicited yet, but it is known that alterations in the metabolite levels have been observed in some degenerative diseases [134]. Additionally, the anthranilate was found to have more pronounced effect on active than on resting rate of respiration. This metabolite (1.25–5 mM) has an effect, in a dose-dependent way, on the respiratory parameters: it dropped state III and respiratory control index using 5 mM glutamate/malate as respiratory substrates. On the other hand, no effect was seen in the presence of succinate or NADH as substrates [86, 135]. These contradictory effects found for ANA can be due to its capability to produce hydroxyl radicals to the 3-HA metabolite, considering that ANA is a substrate to produce it.

### 3.8. Picolinic Acid

Picolinic acid (PIC) is a six-member ring structure and isomer of nicotinic acid, containing five carbon atoms, a nitrogen, and a carboxyl group at position 2. The most widely studied physical characteristic of PIC is its efficient chelator properties; it was first described that this metabolite was an efficient chelating agent for both copper and iron. Later, Suzuki and coworkers described that PIC was also able to chelate other bivalent metals including Ni^2+^, Zn^2+^, Cd^2+^, Pb^2+^, and Cu^2+^ [136]. Therefore, picolinate is an unselective metal ion chelator [137] and also activates macrophages via induction of macrophage inhibitory protein- (MIP-) 1*α* and MIP-1*β*, which is potentiated by simultaneous IFN-*γ* treatment [138]. It also possesses both extracellular and intramacrophage antimicrobial activity against *Mycobacterium avium* [139] and *Candida albicans* [140] and antiviral/apoptotic activity against HVI-1 and Herpes simplex virus-2-infected cells [141]. Additionally, PIC is able to induce synergistically with IFN-*γ*, the expression of nitric oxide synthase in macrophages [142].

Moreover, PIC also has been shown to protect the cholinergic neurons of the nucleus basalis magnocellularis and the nicotinamide adenine dinucleotide diaphorase containing neurons of rat striatum against QUIN-induced neurotoxicity [143, 144]. This protection can be related to the fact that PIC significantly decreases glutamic acid release, evoked by exposure of striatal slices to 1 mM kainate in the presence of calcium. In the absence of external calcium, PIC (100 *μ*M) failed to influence kainic acid-induced release [145]. Additionally, it has been proposed that PIC may act as a glycine agonist at strychnine-sensitive receptors since it was able to reduce the inhibition of firing by glycine in these receptors [146].

However, *α*-PIC chelates Fe^2+^ ions and enhances the hydroxyl radical formation. This effect is attributed to its structure; the two adjacent atoms in the 2-pyridinecarboxylic acid moiety, that is, the nitrogen atom in pyridine ring and the oxygen atom in the carboxyl group, seem to be participating in the chelation of Fe^2+^ ion [147]. Some reports also show the toxic effect of this metabolite since its systemic administration produces alterations in neuronal cell bodies. These alterations developed within selected regions of the brain, as was demonstrated within the hippocampus, substantia nigra, and striatum [148]. Additionally, results indicate that PIC alters cell shape by changing the pattern of distribution of cytoskeletal elements in culture normal rat kidney (NRK) and SV40-transformated NRK cells [149]. All these toxic effects may be related to the hydroxyl radical produced by PIC.

### 3.9. Quinolinic Acid

Quinolinic acid (QUIN), a neuroactive metabolite of the kynurenine pathway, is an agonist of N-methyl-D-aspartate (NMDA) receptor; it has a high *in vivo* potency as an excitotoxin [150]. Free radical generation and oxidative stress are involved in the toxicity induced by QUIN; however it is necessary to have in mind that these mechanisms can be or not dependent of QUIN activity on NMDA receptors. The ROS NMDA receptor-dependent production is promoted by Ca^2+^ entry, which induces the NOS activity and decreases the SOD activity, leading to excess of nitric oxide and superoxide. The interaction between these radicals quickly produces peroxynitrite [151, 152]. Additionally, it has been shown that QUIN can reduce glutathione as well as copper and zinc-dependent superoxide dismutase activity (Cu, Zn-SOD) [153] and induce ROS production, lipid peroxidation, and cell death [154, 155]. Other toxic effects of QUIN through NMDA receptors have been observed like inflammatory events, energetic deficit, behavioral and morphological alterations [150, 156, 157]. It has been shown that depending QUIN levels it can change its activity and toxicity. Several authors have demonstrated the QUIN participation in apoptosis of different cells like oligodendrocytes, neurons, and astrocytes via NMDA-dependent ROS formation. Braidy and coworkers observed that QUIN can act as a substrate for NAD^+^ synthesis at very low concentrations (<50 nM) but can also be a cytotoxic agent at subphysiological concentrations (>150 nM) through the NMDA overactivation, NOS induction, and nitric oxide increase conducing to free-radical damage in astrocytes and neurons. Also, the increased PARP activity leads to NAD^+^ depletion and consequently to cell death [158, 159].

Additionally, Stipek and coworkers (1997) showed that QUIN is able to form complexes with Fe^2+^ and modulate the lipid peroxidation [160]. In phosphate buffer, the QUIN-Fe^2+^ complex enhances the formation of hydroxyl radical via the Fenton reaction, compared to Fe^2+^ ions alone, and also inhibits the auto-oxidation of Fe^2+^ [161]. Further investigation has suggested that the QUIN-Fe^2+^ complex is relatively stable at physiological pH, and although this initiates the generation of hydroxyl radicals, a further QUIN derivative is formed, which enables redox cycling of Fe^2+^ and Fe^3+^ ions, thus maintaining hydroxyl radical formation [162]. The QUIN-Fe^2+^ complex was shown to be also responsible for *in vitro* DNA chain breakage and lipid peroxidation mediated by hydroxyl radicals [79]. Therefore, the generation of reactive oxygen species by QUIN is secondary to the formation and slow pH-dependent autooxidation of QUIN-Fe^2+^ complexes and can be readily prevented by iron chelation [162, 163]. All these evidences suggest that QUIN-Fe^2+^ complexes display significant prooxidant characteristics that could be of concern for QUIN neurotoxicity.

Different ROS scavengers, molecules with antioxidant properties, inducers of activity of antioxidant enzymes, and some pharmacological substances have been tested successfully against QUIN toxicity, showing protection of nervous tissue from oxidative damage induced by QUIN *in vitro* and *in vivo* [164–172].

## 4. Kynurenines Disturbances in Aging and Neurological Diseases

Alterations at the level of kynurenine pathway metabolites and enzymes have been observed in the aging (Table 3) [173, 174] and in several age-associated neuropathological conditions and diseases involving immune activation [175]. However, few studies have investigated changes in tryptophan metabolism with aging. Upregulation of tryptophan-KYN metabolism has been reported in older individuals (72–93 years of age) as compared with younger adults (34–60 years of age) [176]. A study concerned with the formation of UV filters in the human lens, which are formed from L-tryptophan through the KP, observed the highest levels of kynurenine in lenses (postmortem) from young people, below the age of 20 years, and lowest levels were detected in lenses of 80 years of age or older, suggesting that the protective effect of the metabolite against UV damage is reduced with the advancing of age [177]. In a study in rats was found a significant decrease in liver TDO activity with age [178], while another showed anomalous tryptophan catabolism, partly because of vitamin B6 and nicotinamide deficiency [179]. In this context, Braidy and coworkers [180] showed a significant decrease in TDO activity with age progression in the brain, liver, and kidney of female rats. Additionally, it was observed a significant increase in IDO brain activity with age, which is consistent with the observed that there is age-dependent increase of KYN in brain. This raising in available KYN is probably enough to explain the described age-dependent increase in KYNA, PIC, and QUIN. These observations may reflect adaptive changes related to the aging process in immune activity within the brain [178]. Under this perspective, aging is associated with the chronic, low grade, Th-1 type inflammation, in which IFN-*γ*, a potent proinflammatory cytokine and an inducer of IDO, is involved [181].

In another study related to enzymatic variations with age, IDO activity was measured. In the group of rats aged 2-3 months, the highest specific activity was observed in the small intestine and the lowest in the lungs and kidneys, whereas at 12 months of age the highest IDO activity was found in the brain, and kidneys presented the lowest activity. At 18 months, IDO returned to be more elevated in the small intestine. At 12 months old the values of IDO in tissues varied slightly, while at 18 months similar activities were found between lungs and brain and between the small intestine and kidneys. In relation to age, IDO specific activity declined in the small intestine, after 2-3 months of age [182].

Additionally, Moroni and coworkers [183] described a similar increase of KYNA levels in the aging rat brain. The brain concentration of KYNA was extremely low during the first week of life; then it increased at 3 months and a high raise was observed at 18 months of age, in accordance with the data of Finn and coworkers [184] and Gramsbergen and coworkers [185]. A positive relationship between CSF KYNA levels in humans and ageing has also been reported [186]. Elevated KYNA metabolism may be involved in the hypofunction of the glutamatergic and/or nicotinic cholinergic neurotransmission in the CNS of ageing humans [186]. Additionally, the increases of KYNA levels could underlie cognitive decline found in the aging.

Moreover, QPRTase activity in the brain is reduced with ageing, in parallel with an age-related increase in QUIN [180, 183]. An excessive accumulation of QUIN in brain tissue can induce a cytotoxic cascade within the CNS [187]. Increased QUIN content in the aging rat brain also suggests that the activity of the enzyme leading to the synthesis of QUIN (3-HAO) may also increase in the brain with advancing age [178].

These changes in metabolites and enzymes of KP are related to reports that show a decline in NAD^+^ levels and an increase in oxidative markers [188], suggesting a strong link between these factors in longevity which allow to propose KP as a therapeutic target to modulate free radicals and restore NAD^+^ levels.

On the other hand, neurodegenerative diseases are related to disturbances of the mitochondrial function, oxidative stress, and alterations in kynurenines levels [189, 190]. In this study we have described the redox activity of the kynurenines and how the KP can be modulated by the environment; however, their production in several pathologies can be more difficult to clarify since many factors converge and can change the cellular environment.

Alterations in the kynurenines metabolism can be due to alteration of energetic metabolism, oxidative damage, and inflammation, affecting the cellular function. Its relevance can be viewed under pathological conditions [86, 134, 135, 189, 191–194].  Table 4 summarizes changes in the KP metabolites found in different neuropathologies.

## 5. Modulation of KP and Its Implications in the Intracellular NAD^+^ Levels

Recent studies have focused on the possible effects modulating the KP. The main strategies to follow are (1) tryptophan supplementation, (2) the use of inhibitors of the KP's enzymes, and (3) the use of analogues of KP metabolites, as KYNA. The first strategy has been widely studied considering that Trp, besides from being a precursor of kynurenines, is also of serotonin and melatonin. Trp supplementation has been used as a helpful therapy to treat behavior problems in animals since a low-protein diet supplemented with Trp helped in managing canine aggression problems [195]. Nevertheless the specific mechanism by which Trp acts in this way is still unclear but might be also due to the neuroactive metabolites of KP. In this context, Ciji et al. found increased serum cortisol levels and decreased serum testosterone and estradiol levels in fish exposed to nitrite; these effects were prevented by vitamin E and tryptophan diet; however, the benefits effect of vitamin E were due to its antioxidant characteristics, but the effect of Trp was unclear. Nonetheless, its protective effect may be not only the result of its own redox properties but also due to the metabolites with antioxidant properties produced by Trp degradation [196]. Moreover, a study showed that in healthy women under a rich diet in Trp increased the urinary excretion of KYN, ANA, KYNA, 3-HK, 3-HA, and QUIN [197], which under certain circumstances can be toxic. In fact, it has been shown that the excessive Trp supplementation would aggravate or would induce autoimmune diseases [198] due to the metabolites produced during its metabolism. The Trp supplementation can also affect intracellular NAD^+^ levels.

Braidy and coworkers have recently shown that 3-HA, 3-HK, and QUIN can promote NAD^+^ synthesis at concentrations below 100 nM in human primary astrocytes and neurons. However, these metabolites at concentrations >100 nM decreased intracellular NAD^+^ levels and increased extracellular LDH activity in both primary human astrocytes and neurons [158]. The vulnerability showed in human cerebral neurons may be due to the fact that the neurons can take up exogenous QUIN but can only catabolize a small amount [199] since QPRT is rapidly saturated. These events, depending on the kynurenines concentration, need to be taken into consideration since the biosynthesis of NAD^+^ is vital to the maintenance and ongoing cell viability of all of them.

The inhibition of IDO, KMO, and QPRT represents an important pharmacological target, since the kynurenines are involved in many neurodegenerative diseases. Several experimental models have been used to test some inhibitors of specific KP's enzymes. The inhibition of IDO and QPRT activities with 1-methyl-L-tryptophan and phthalic acid, respectively, resulted in reduction of intracellular NAD^+^ and cell viability, in both astrocytes and neurons; however, these effects are higher in neurons than astrocytes, suggesting that changes in KP metabolism have a greater effect on the neuronal population compared to glial cells. It is noteworthy that in a mouse model for multiple sclerosis the IDO inhibition aggravated the disease progression, denoting that IDO inhibition exacerbated the disease [200]. This could be related to the fact that IDO inhibition reduces NAD^+^ synthesis and therefore promoting cell death.

Following the same line, Blight and coworkers observed that treatment with 4-chloro-3-hydroxyanthranilate, a synthetic inhibitor of 3-hydroxyanthranilic acid oxidase, was able to reduce QUIN production and functional deficits following experimental spinal cord injury in guinea pigs [201]. More recently, it has been showed that the KMO inhibitor, 3,4-dimethoxy-N-[4-(3-nitrophenyl)-thiazol-2-yl]-benzenesulfonamide (Ro61-8048) [202] prevents ataxia and death in mice infected with the malaria parasite *Plasmodium*. This protection was associated with the elevated levels of KYNA and ANA [203]. Additionally, Campesan and coworkers showed the first evidence that inhibition of KMO and TDO activity protects against a transgenic *Drosophila melanogaster* model of Huntington disease [204, 205]. According to this subject, the oral administration of JM6, a novel prodrug inhibitor of KMO, avoided behavioral deficits and synaptic loss and raised KYNA levels in well-established genetic mouse models of Alzheimer [206]. Actually, whereas KMO inhibition leads to brain 3-HK and QUIN reduction, this may provide benefits in neurodegenerative diseases [203, 207–212]; the blockade of KAT II brings about a decrease in brain KYNA but can be related to cognition-enhancing effects [213, 214].

Further studies are necessary to explore whether prolonged manipulations of both KP metabolism arms have diverse consequences and which experimental models could be the best strategy because KYNA promotion can not only be an effective target just in some neurotoxic models, those that display a great excitotoxic damage, but can also promote NAD^+^ depletion and in a prolonged time could lead to cell death.

## 6. Concluding Remarks

In recent years, different groups and researchers have investigated the redox properties of KP metabolites; however, due to the dual effects of these metabolites and the high degree of modulation of the KP (inflammatory cytokines, metals, pH, and redox environment), is complex it try to establish a precise mechanism by which cellular alterations can be produced. What we do know is that these metabolites must have a physiological activity and a great impact on aging and especially in pathological conditions, processes in which are also altered factors that regulate the production of these kynurenines. The precise degree of involvement of these events constitutes a fertile line of research to explore in the next years.

## Figures and Tables

**Table 1:** Kynurenine pathway enzymes and their positive and negative regulators.

Enzyme	Reaction catalyzed	Positive regulators	Negative regulators
Tryptophan 2,3-dioxygenase	L-Trp + O_2_/O_2_^∙−^ → N-formyl-L-kynurenine	Melatonin, H_2_O_2_ [215]. O_2_^∙−^ [216].	3-HK, KYNA, XA, NADH [217]. Cu^2+^ [218]. Superoxide dismutase (SOD) [216].

Indolamine 2,3-dioxygenase	L-TRYP + O_2_/O_2_^∙−^ → N-formyl-L-kynurenine	O_2_^∙−^ IFN-*α*/*β*/*γ*, lipopolysaccharide, hiperoxia [12, 219].	SOD [220]. NO [221]. H_2_O_2_, IL-4 [12].

Formamidase	N-formyl-L-kynurenine + H_2_O → formate + L-KYN	H_2_O, ascorbic acid, arginine, L-TRYP [222].	ANA [223]. 3-HK, Mn^2+^ [222].

Kynureninase	L-KYN + H_2_O → ANA + L-alanine	H_2_O, 3-HK [224].	

Kynurenine aminotransferases	L-KYN + 2-oxoglutarate/pyruvate → KYNA + L-glutamate	2-Oxoglutarato, pyruvate, 2-aminoadipate, pyridoxal 5′-phosphate [225, 226].	Glutamine, L-cysteine, 3-HK, L-phenylalanine, L-tryptophan, L-aspartate [191, 227–229].

Kynurenine 3-monooxygenase	L-KYN + NADPH + O_2_ → 3-HK	NADPH, O_2_, FAD, NADH, inflammatory stimulus [27, 230, 231].	ANA, XA, Cl^−^, pyridoxal 5′-phosphate [28, 232].

3-Hydroxyanthranilic acid 3,4-dioxygenase	3-HA + O_2_ → 2-amino-3-carboxymuconate-6-semialdehyde	O_2_, Fe^2+^ [233].	Zn^2+^ [233].

2-Amino-3-carboxymuconate-6-semialdehyde decarboxylase	2-amino-3-carboxymuconate -6-semialdehyde → 2-aminomuconic-6-semialdehyde + CO_2_	KYNA, PIC, QUIN [234].	Zn^2+^, Fe^2+^ [234, 235].

Quinolinic acid phosphoribosyltransferase	QUIN + 5-phospho-*α*-D-ribose 1-diphosphate → NAD^+^ + diphosphate + CO_2 _	Mg^2+ ^[236, 237].	ATP, Cu^2+^ Fe^2+^, Fe^3+^, Zn^2+^ [238].

**Table 2:** Kynurenines with redox behavior.

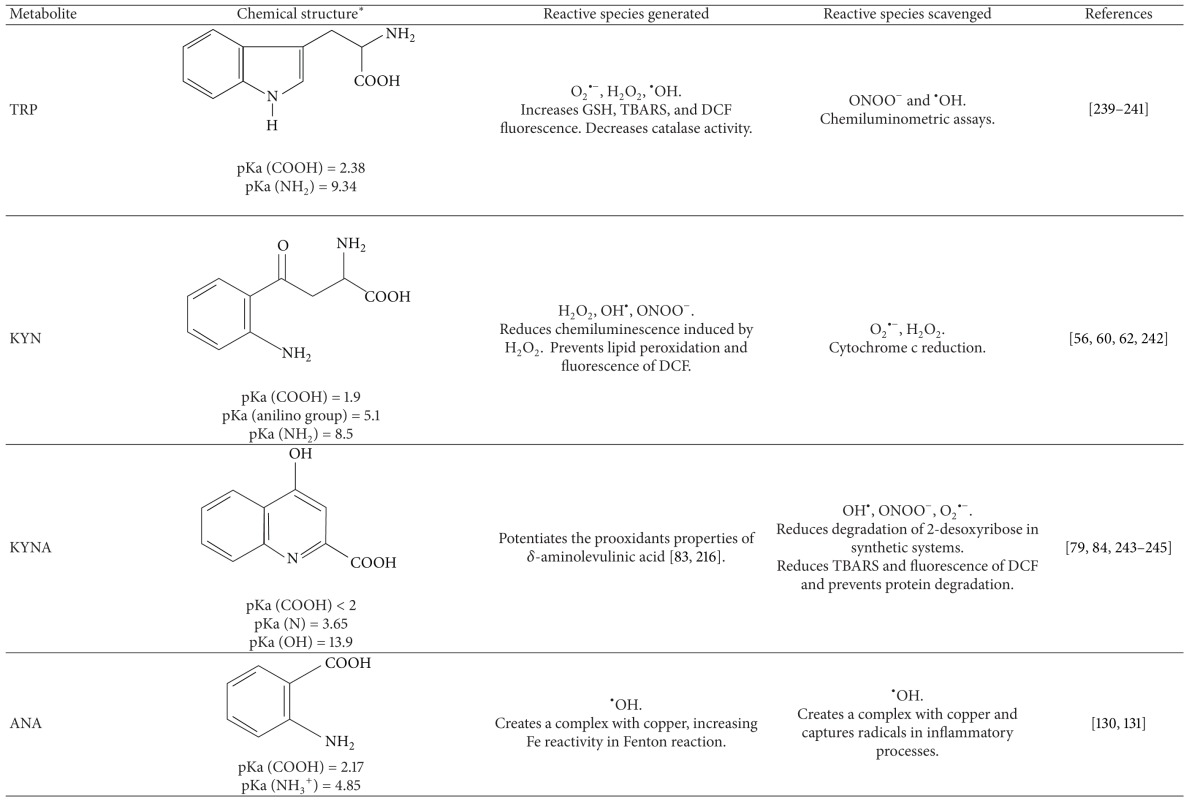 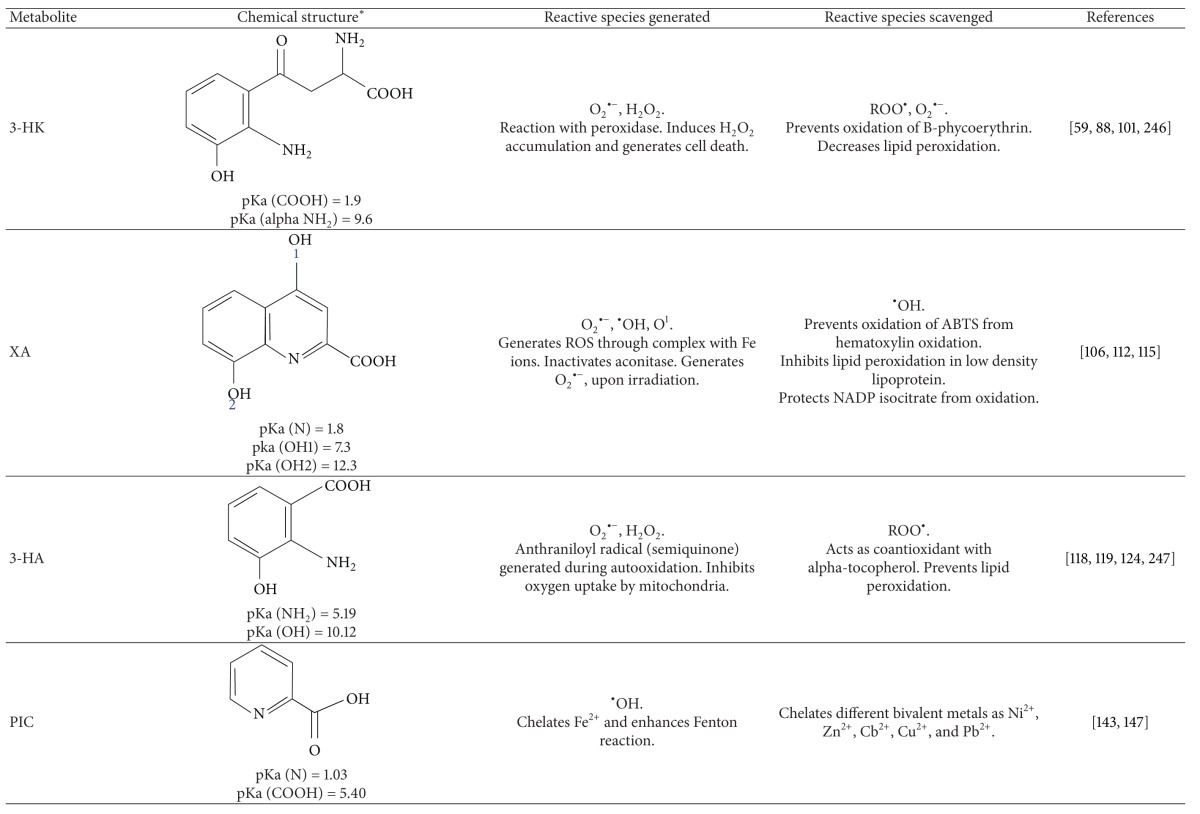 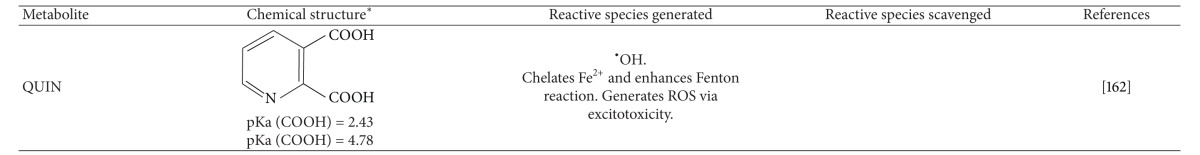

*The chemical structures were built with the program ACD/ChemSketch Freeware (http://www.acdlabs.com/resources/freeware/chemsketch/).

**Table 3:** Changes in kynurenine pathway's metabolites and enzymes with the age in rats [178, 180, 185, 248].

Metabolite/enzyme	Brain	Liver	Kidney
TRP	↓	↓	↓

TDO	↓	↓	↓

IDO	↑	↓	↓

KYN	↑	↓ at 12 months No changes at 24 months	↓ at 12 months ↑ at 24 months

KATs	↑	No changes	↑

KYNA	↑	No changes	↑

KMO		↓	↓

Kynureninase		↓	↓

3-Hydroxyanthranilate 3,4-dioxygenase		↑	↑

Aminocarboxymuconate-semialdehyde decarboxylase (ACMSD)		↑	↑

QPRT	↓	↓	↑

QUIN	↑	↑	↓ at 12 months No changes at 24 months

PIC	↑	↑	↓ at 12 months No changes at 24 months

**Table 4:** Alterations in kynurenines levels in neurodegenerative diseases.

Disease	Metabolite	Sample	Reference
Alzheimer disease	(i) ↑ TRP/KYN ratio	Plasma	[249]
	(ii) ↑ KYNA levels and KAT I activity	Putamen and caudate nucleus	[192]
	(iii) ↓ KYNA levels	CSF and plasma	[250, 251]
	(iv) ↑ 3-HK	Serum	[252]

Huntington disease	(i) ↑KYNA and 3-HK levels	Neostriatum and cortex in early-stage HD patients	[253]
	(ii) ↓ 3-HK and 3-HA		[254]

Parkinson disease	(i) ↓ KYNA	Frontal cortex, putamen, and SNpc of patients with PD, CFS	[255, 256]
	(ii) ↑ 3-HK		

Schizophrenia	(i) ↓ KMO and 3-HAO	Prefrontal cortex	[257, 258]
	(ii) ↑ L-KYN and KYNA		

Depression	(i) ↓ TRP	Plasma	[259–262]
	(ii) ↑ KYN		
	(iii) ↑ IDO		
